# DoTools: a cross platform framework to streamline common single cell workflows

**DOI:** 10.1093/bioadv/vbag098

**Published:** 2026-04-29

**Authors:** Mariano Ruz Jurado, David Rodriguez Morales, Lukas Zanders, Elijah Genetzakis, Stefanie Dimmeler, David John

**Affiliations:** Institute of Cardiovascular Regeneration, Goethe University Frankfurt, Theodor-Stern-Kai 7, Frankfurt am Main, Hessia, 60590, Germany; German Centre for Cardiovascular Research (DZHK), Frankfurt am Main, 60590, Germany; Cardiopulmonary Institute, Goethe University Frankfurt, Frankfurt am Main, 60590, Germany; Institute of Cardiovascular Regeneration, Goethe University Frankfurt, Theodor-Stern-Kai 7, Frankfurt am Main, Hessia, 60590, Germany; German Centre for Cardiovascular Research (DZHK), Frankfurt am Main, 60590, Germany; Cardiopulmonary Institute, Goethe University Frankfurt, Frankfurt am Main, 60590, Germany; Institute of Cardiovascular Regeneration, Goethe University Frankfurt, Theodor-Stern-Kai 7, Frankfurt am Main, Hessia, 60590, Germany; German Centre for Cardiovascular Research (DZHK), Frankfurt am Main, 60590, Germany; Clinic for Cardiology, University Hospital Frankfurt, Frankfurt am Main, 60590, Germany; Institute of Cardiovascular Regeneration, Goethe University Frankfurt, Theodor-Stern-Kai 7, Frankfurt am Main, Hessia, 60590, Germany; German Centre for Cardiovascular Research (DZHK), Frankfurt am Main, 60590, Germany; Cardiopulmonary Institute, Goethe University Frankfurt, Frankfurt am Main, 60590, Germany; Institute of Cardiovascular Regeneration, Goethe University Frankfurt, Theodor-Stern-Kai 7, Frankfurt am Main, Hessia, 60590, Germany; German Centre for Cardiovascular Research (DZHK), Frankfurt am Main, 60590, Germany; Cardiopulmonary Institute, Goethe University Frankfurt, Frankfurt am Main, 60590, Germany; Institute of Cardiovascular Regeneration, Goethe University Frankfurt, Theodor-Stern-Kai 7, Frankfurt am Main, Hessia, 60590, Germany; Cardiopulmonary Institute, Goethe University Frankfurt, Frankfurt am Main, 60590, Germany

## Abstract

**Motivation:**

Throughout the years, single-cell RNA sequencing (scRNA-seq) has become a standard approach for characterising transcriptomic changes associated with diseases and other biological conditions. However, the rapid expansion of tools and algorithms developed in various programming languages has made single-cell data analysis increasingly complex. In particular, integrating multiple tools into a single workflow often demands substantial learning time and coding expertise.

**Results:**

To address these challenges, we developed DoTools, a unified framework for R/Bioconductor and Python/PyPI that simplifies the integration of third-party tools such as scVI, CellTypist, and CellBender into standard pipelines like Seurat, SingleCellExperiment and Scanpy. DoTools provides advanced cross-language wrappers and visualisation utilities to streamline data preprocessing, quality control, cell type annotation, and downstream analysis, while implementing best practices in scRNA-seq analysis regardless of the computational language. Its modular design and compatibility with widely used bioinformatics environments makes it accessible and valuable to both novice and experienced data scientists.

**Availability and implementation:**

DoTools is freely available for R and Python at Bioconductor and PyPI (https://bioconductor.org/packages/release/bioc/html/DOtools.html and https://pypi.org/project/DoTools-py/), the developmental versions of DoTools are maintained on GitHub (https://github.com/MarianoRuzJurado/DoTools and https://github.com/davidrm-bio/DoTools_py).

## Introduction

Advances in omics technologies, particularly single-cell RNA sequencing (scRNA-seq), have provided an unbiased framework to study cellular heterogeneity, shifts in cell type composition, and the dynamics of cells in different organs and tissues (Luecken and Theis 2019). The workflow for generating scRNA-seq data can be summarised into four steps: (i) sample preparation, (ii) library preparation, (iii) sequencing, and (iv) bioinformatic analysis. As approaches to sample preparation, and barcoding techniques are refined, these technologies are simultaneously evolving side-by-side leading to an improvement in data quality and enabling larger datasets.

This has allowed researchers to build disease specific atlases (Nicin *et al.* 2022) to understand the underlying mechanisms for pathological conditions. At the same time, the growing number of computational methods for preprocessing, normalisation, integration, and cell annotation has complicated the development of efficient and reproducible analysis pipelines.

Popular frameworks such as Seurat (Hao *et al.* 2021) or Scater (McCarthy *et al.* 2017) in R and Scanpy (Wolf *et al.* 2018) in Python provide a foundation for scRNA-seq analysis. However, cross-platform availability of individual methods is still limited. Additionally, the incorporation of external tools and frameworks can be challenging for novice users.

DoTools aims to provide standardised wrappers that extend existing single-cell analysis workflows (Table 1), enabling streamlined data processing, format conversion, and complementary visualisation while adhering to established single-cell best practices (Luecken and Theis 2019). It simplifies the often lengthy and complex workflows without losing adaptability. DoTools was built in a modular framework, allowing the expansion or modification of the workflow to include and adapt the methods based on community recommendations. DoTools is available as an R/Bioconductor and Python/PyPI package under MIT license and is actively maintained in accordance with Bioconductor’s long-term support and maintenance requirements.

**Table 1:** Comparison of DoTools with commonly used single-cell analysis frameworks.

	Scanpy	Seurat	scFlow	DoTools
**Platform**	Python	R	R	Python/R
**Ambient RNACorrection**	–	–	emptyDrops	CellBender
**Filter low quality genes and cells**	thresholds	thresholds	tresholds	thresholds and quantiles
**Doublet detection**	Scrublet	–	DoubletFinder	scDblFinder, Scrublet, DoubletDetection
**Integration**	Ingest, Harmony Scanoramam mnn, BBKNN	CCA, Harmony, JointPCA, RPCA	Liger	scVI, scANVI, Harmony, Scanorama, CCA, BBKNN, RPCA, JointPCA
**Semi-automatic annotation**	–	–	monocle, EWCE	Celltypist, scANVI
**Differential gene analysis**	Wilcoxon, t-test, logistic regression	DESeq2, Mast, Wilcoxon, LikelihoodRatio, roc, poisson, logistic regression, t-test	Mast, Limma	Wilcoxon, (Samplewise-) t-test, EdgeR, DESeq2, Mast, LikelihoodRatio, poisson, logistic regression
**Data visualisation**	UMAP, Dotplot, Violinplot, Heatmap, Tracksplot	UMAP, Ridgeplot, Dotplot, Violinplot, Heatmap	UMAP, Violinplot	UMAP, Dotplot, Violinplot, Boxplot, (log2fc-)Heatmap, Barplot, Lineplot
**Statistical plots**	–	–	–	**✓**
**Gene ontology**	**✓**	**✓**	**✓**	**✓**
**Cell composition changes**	–	–	**✓**	**✓**

## Description of DoTools

**Figure 1:**
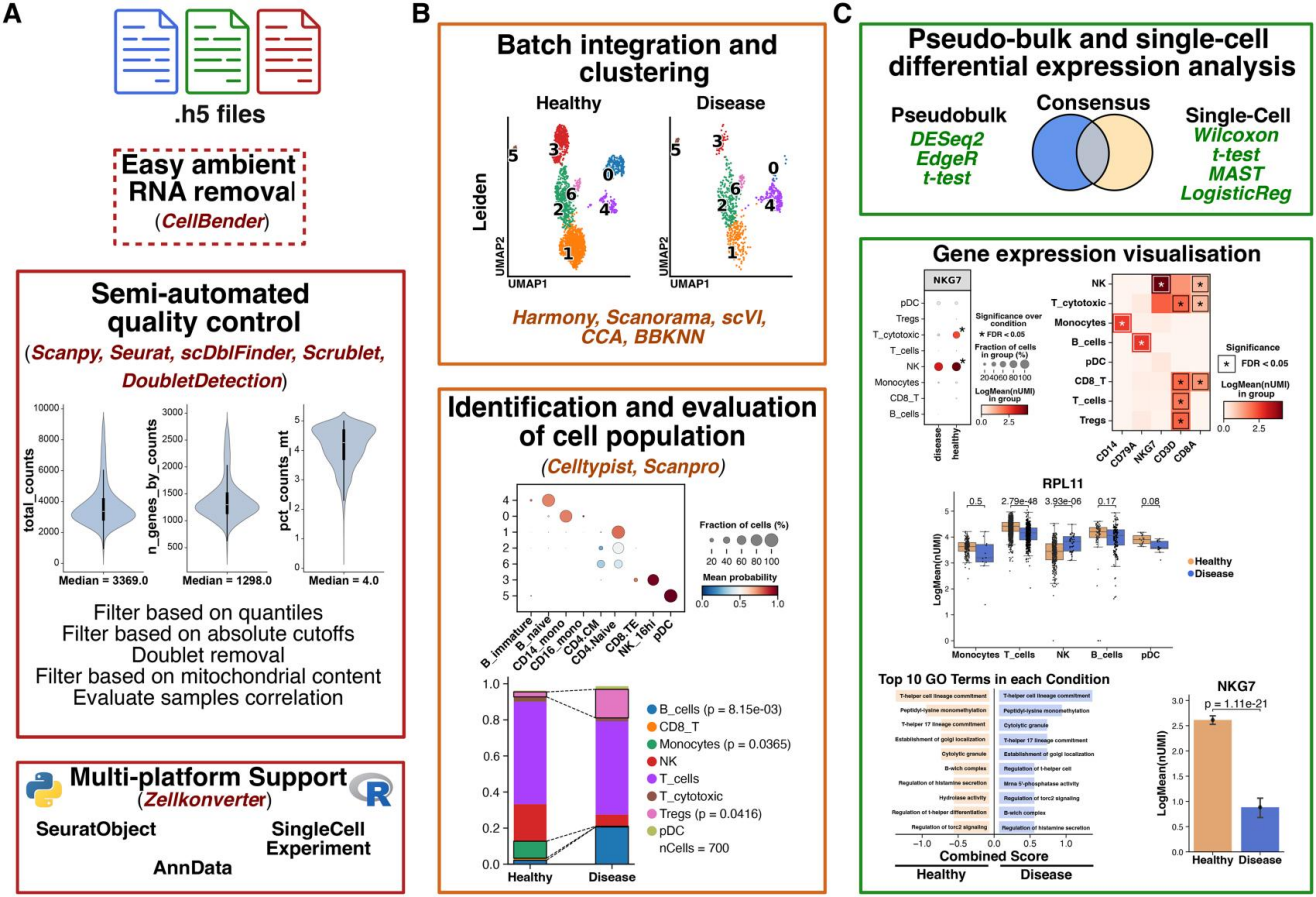
Workflow for DOTools package. (A) The initial input consists of H5 files generated by alignment tools like CellRanger or STARsolo. As an optional step, ambient RNA can be removed with CellBender. Next, a basic quality control is performed. The package allows the conversion between AnnData, SeuratObject, and SingleCellExperiment. (B) After the quality control, different integration methods can be used followed by clustering and supported cell type annotation. Significant changes in cell populations can be identified. (C) Differential gene expression analysis using pseudobulked or single-cell approaches can be used. The package offers different visualisation with the option to include statistics on them. The methods or packages that are used or available for the different steps are highlighted using the same color as the square in each section.

DoTools provides a workflow for data analysis, and visualisation for scRNA-seq data (Fig. 1). The package provides implementations in both R and Python, enabling seamless cross-platform integration. It is freely available at Bioconductor (https://bioconductor.org/packages/release/bioc/html/DOtools.html) and from PyPI (https://pypi.org/project/DoTools-py/), ensuring accessibility to a wide range of users. Detailed tutorials and examples can be found in the extended documentation (https://dotools-py.readthedocs.io/en/stable/ for Python and https://marianoruzjurado.github.io/DOtools/ for R). The DoTools pipeline was tested on Linux, macOS, and Windows systems. For a standard single-cell experiment we recommend a machine with at least 16GB of RAM memory and at least 5 CPUs. The scalability of DoTools was successfully tested on large dataset with over 150k cells (Ruz Jurado *et al.* 2025; Rodriguez Morales *et al.* 2025).

## Datasets

All examples in the package use 3’ sequencing data using the 10X Genomics Chromium platform, which is publicly available from the 10X Website, generated either from peripheral blood mononuclear cells (PBMCs) from one healthy donor or extranodal marginal zone B-cell Tumor from one donor.

## Input files

The DoTools package requires unprocessed H5 files generated from any alignment tool like CellRanger or STARsolo, which are subsequently used for initial quality control (Fig. 1A). Alternatively, preprocessed data can be supplied as an AnnData, SeuratObject, or SingleCellExperiment object. Methods to allow conversion between the three data structures are available allowing easy cross-platform conversion.

## Data processing

The initial input is an unprocessed H5 file. Depending on the properties of the experiment, the user can optionally correct for ambient RNA via CellBender (Fleming *et al.* 2023) (Fig. 1A). This step is important for droplet-based single-cell assays, where background noise counts can be generated causing nonzero counts in cell-free droplets and off-target gene expression, potentially introducing batch effects. After successful ambient RNA removal an H5 file is generated, which can be used directly in the next steps.

To perform the initial quality control of the dataset, we offer one main function *DO.Import (R) or do.pp.importer_py* (Python). These functions enclose all the community recommended quality control steps (Luecken and Theis 2019) for scRNA-seq: including removal of lowly expressed genes and low quality cells based on different parameters such as the mitochondrial content or the unique molecular identifiers (UMI) counts. Additionally, neotypic doublets, which are generated from cell types with different transcriptomic profiles can also be identified and removed using various methods, such as DoubletDetection (Gayoso and Shor 2025), Scrublet (Wolock *et al.* 2019), and scDblFinder (Germain *et al.* 2021) (Table 1). Besides neotypic doublets, scRNA-seq assays can also produce embedded doublets, which arise from cell types with similar transcriptomic profiles. To remove these cells, we offer the possibility to filter cells based on UMI counts using quantiles or absolute thresholds. During the processing of the samples, diagnostics plots will be generated including violin plots and a table recording changes in the number of cells and genes after each step in the quality control. After the initial preprocessing, an AnnData, SeuratObject or SingleCellExperiment object will be generated (Fig. 1A).

Following quality control, normalisation is performed using the shifted logarithm transformation that has been shown to perform well stabilizing variance for subsequent dimensionality reduction and identification of differentially expressed genes. Next, an important step in scRNA-seq analysis is the batch correction. Several methods are available, which can easily be run using the function *DO.Integration* (R) or *do.tl.integrate_data* (Python). Among the available methods we offer Harmony (Korsunsky *et al.* 2019), scVI (Lopez *et al.* 2018), BBKNN (Polański *et al.* 2020), Scanorama (Hie *et al.* 2024), and CCA (Hao *et al.* 2021) (Fig. 1B). Since the choice of the integration method depends on the nature of the dataset, we additionally offer different methods to evaluate how well they removed batch effects. A tutorial showing how well the different integration methods work is available in the documentation. Benchmarking of the different integration methods, using DoTools on a dataset with ∼65k cells on a machine with 10 CPUs showed variation in runtime. Methods like CCA and scVI took the longest with 101 and 45 minutes, respectively while the rest of the methods took 2 minutes (Harmony and BBKNN) and 1 minute (Scanorama). Next, to facilitate cell type annotation, DoTools provides a dedicated function that can be invoked using *DO.Celltypist* (R) or *do.tl.auto_annot* (Python). These methods are built on Celltypist (Domínguez Conde *et al.* 2022), a framework employing regularised linear models optimised by stochastic gradient descent to infer cell identities using models trained on diverse tissues and organs.

Differential gene expression analysis can be performed using two different modes: pseudobulk or single-cells (Fig. 1C). To perform DGE analysis on the pseudobulk level, different methods are available including DESeq2 (Love *et al.* 2014), edgeR, and t-test (Robinson *et al.* 2010). On the other hand, DGE on the single-cell level can be performed using Wilcoxon, t-test, MAST (Finak *et al.* 2016), and LogisticRegression models. Besides running these tests individually, we offer the possibility to run multiple tests, generating a consensus summary, which can be performed using *DO.MultiDGE* (R) or *do.tl.rank_genes_consensus* (Python). These methods allow to perform the DGE analysis for all cell types at the same time.

## Data visualisation

**Figure 2:**
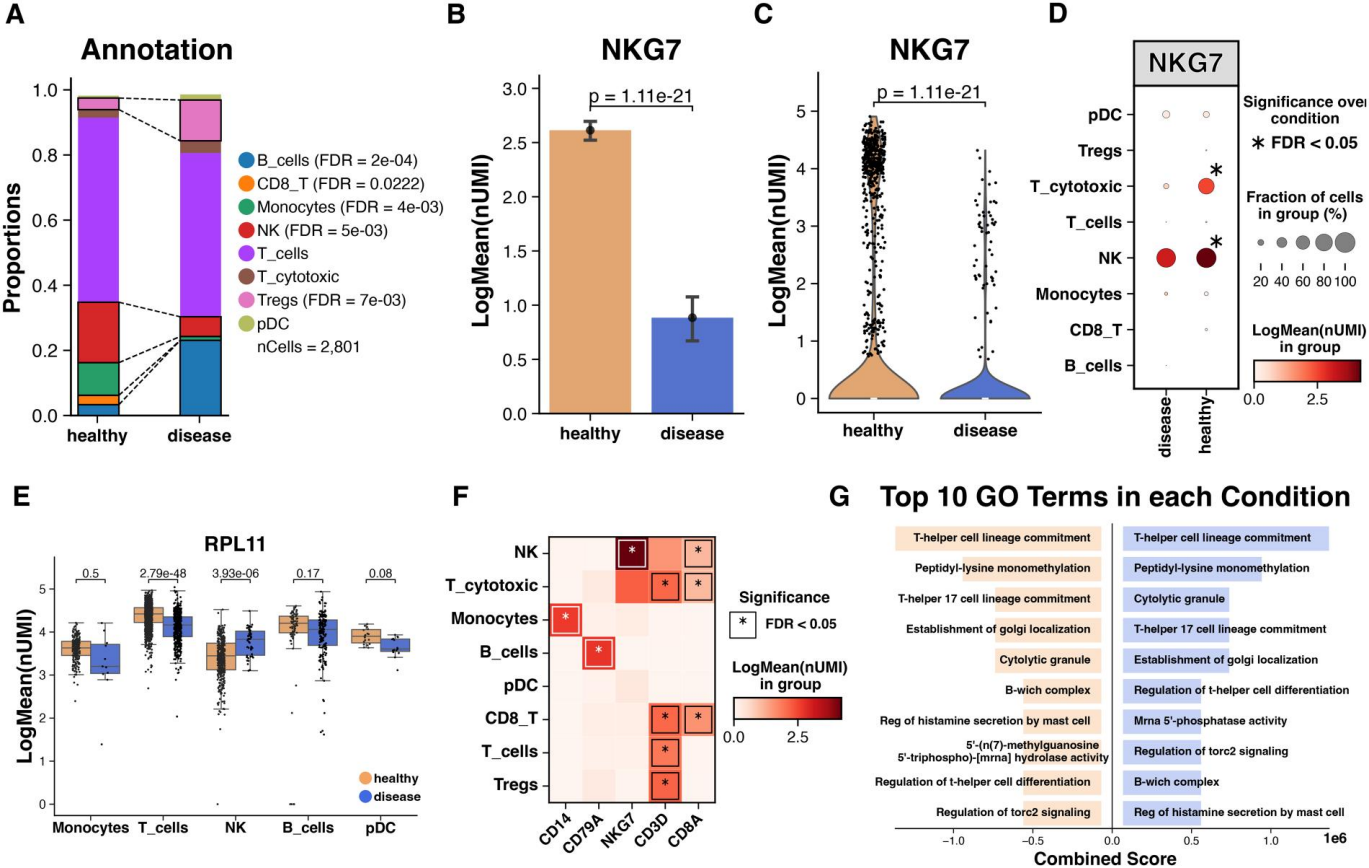
Representative visualisation plots. (A) Mean cell type proportions across conditions (significant differences are marked with discontinued lines). (B–D) Mean and distribution of the NKG7 expression across groups. (E) Distribution of RPL11 expression across cell types and conditions. (F) Mean expression of features across cell types, hierarchical clustering was performed across the x and y axis. (G) Top gene ontology terms enriched in each condition sorted by the combined score estimated by EnrichR. Significance was tested using T-test in (A) and Wilcoxon Test in (B–F).

In addition to the data processing workflow, DoTools offers advanced and customisable visualisation methods. For example, after cell type identification and annotation, changes in the cell type population between conditions can be evaluated with *DO.CellComposition* (R) or *do.pl.cell_composition* (Python) (Fig. 2A). This method can be used to visualise changes in cell type populations across conditions, with significant changes indicated with a discontinued line and the *P*-value in the legend. To compare the expression of specific features between groups, the user can use barplots, violinplots, dotplots, boxplots, and heatmaps (Fig. 2B–F). These methods also allow the incorporation of statistical significance, which is currently not possible with any other framework.

A common workflow after performing DGE analysis is to perform gene ontology analysis. To visualise the top significant terms across two conditions, we also provide the *DO.SplitBarGSEA* (R) and *do.pl.split_bar_gsea* (Python) methods (Fig. 2G).

## Conclusions

The DoTools package provides a user-friendly and cross-platform customisable workflow that allows users to process single-cell RNA sequencing (3′ and 5′) and generate publication-ready graphs of complex data without the need for extensive programming expertise. The methods available can be adapted to other sequencing platforms, such as spatial transcriptomics; however, support is currently limited, and future releases will further improve adaptability. We believe that DoTools will be a valuable resource for the single-cell research community, offering a scalable and extensible foundation for scRNA-seq analysis.

## Data Availability

The data available in this paper is publicly available in the 10X Website (https://www.10xgenomics.com/datasets) and can be downloaded with the DoTools package. The code used to generate the figures is available on Github (https://github.com/davidrm-bio/DoTools_Manuscript).
